# Retrospective transcriptomic analysis indicates temporal dysregulation of mitochondrial genes and metabolic pathways after volumetric muscle loss injury

**DOI:** 10.14814/phy2.70612

**Published:** 2025-11-02

**Authors:** David L. Miller, Sarah M. Greising, Eugene F. Douglas, Jarrod A. Call

**Affiliations:** ^1^ Regenerative Bioscience Center University of Georgia Athens Georgia USA; ^2^ School of Kinesiology University of Minnesota Minneapolis Minnesota USA; ^3^ Pharmaceutical Sciences University of Georgia Athens Georgia USA; ^4^ Department of Physiology & Pharmacology University of Georgia Athens Georgia USA

**Keywords:** mitochondria, muscle injury, muscle recovery, muscle regeneration, secondary data analysis

## Abstract

Volumetric muscle loss (VML) injury results in the irrecoverable loss of muscle mass and strength and alters the metabolic capacity of the remaining muscle tissue. The primary objective of this retrospective study was to leverage existing RNA‐seq datasets to investigate mitochondria and metabolic transcriptome changes after VML injury. The datasets were extracted from publicly available sources and included a bulk RNA‐seq dataset (*Rattus norvegicus*) and a single‐cell RNA‐seq dataset (*Mus musculus*) that combined provided a transcriptional landscape out to 42 days post‐injury (dpi). The Broad Institute's MitoCarta3.0 database was used to identify mitochondrial‐associated genes and pathways for the analysis. There was a robust downregulation of genes in the bulk RNA‐seq dataset out to 28 dpi. Gene set enrichment analysis revealed that these genes contributed to oxidative phosphorylation, fatty‐acid oxidation, and carbohydrate metabolism. A changing metabolic transcriptional landscape was evident in the single‐cell RNA‐seq dataset as several cell types (e.g., satellite cells, macrophages, and fibro‐adipogenic cells) had upregulated gene sets (e.g., oxidative phosphorylation) that switched to downregulated after 14 dpi. Results from this study complement physiological studies that report dysfunctional mitochondrial bioenergetics, particularly for carbohydrate and free‐fatty acid carbon sources, both immediately and chronically after VML injury. These findings also provide targets for monitoring the success of future interventions or directly manipulating in attempts to improve whole‐muscle metabolic function.

## 1 INTRODUCTION

Volumetric muscle loss (VML) injury results in the irrecoverable loss of skeletal muscle mass and contractile capacity due in part to a loss of native regenerative elements required for successful skeletal muscle regeneration (e.g., extracellular matrix and satellite cells) (Caldwell et al., 1990; Corona et al., 2016; Lefaucheur & Sébille, 1995; Lepper et al., 2011) and a cellular environment unconducive to cellular repair (Das et al., 2020; Larouche et al., 2023). Cellular environments that are uniquely affected by the VML injury, in comparison to contraction/myotoxic/chemical‐induced injuries, include those associated with inflammation, fibrotic tissue deposition, vascularization, and myogenesis. For example, inflammatory cells such as natural killer (NK) cells and neutrophils demonstrate a prolonged tissue residence following VML injury and macrophages maintain their pro‐inflammatory polarization longer (Hymel et al., 2023; Larouche et al., 2022; Lokwani et al., 2024; Ngo et al., 2024; Sadtler et al., 2017). The lengthened inflammatory phase after VML injury stimulates fibro‐adipogenic progenitor (FAP) cells to take on a fibrotic phenotype and generate connective tissue within the muscle (Hoffman et al., 2022; Larouche et al., 2018; Lemos et al., 2015). This pathophysiology is underscored by the molecular biology of the cells that comprise the VML‐injured muscle, and the field has only just begun to interrogate these transcriptional changes after VML injury.

To better understand transcriptional changes after VML injury, we focus on two recently published transcriptomic studies conducted in rodents (Aguilar et al., 2018; Larouche et al., 2022). Exceptional features of both studies include a time‐from‐injury time course study design, hindlimb surgical ablation injury model, and appropriate physiological controls. The commonalities between the two studies are contrasted by the transcriptomic approach, wherein one study conducted in rats utilized a bulk RNA‐seq approach (Aguilar et al., 2018) and the other study conducted in mice utilized a single‐cell RNA‐seq approach (Larouche et al., 2022). The bulk RNA‐seq rat study noted that transcriptome pathway analysis was dominated by gene sets relating to fibrotic remodeling of the extracellular matrix, collagen deposition, fibril organization, and cell adhesion accompanied by sustained Wnt and TGF‐β signaling. Inflammatory gene responses supported the notion that an enduring inflammatory phase after VML injury contributes to the dysregulated transcriptional changes (Aguilar et al., 2018). The single‐cell RNA‐seq mouse study explored cellular heterogeneity in addition to transcriptome pathway analysis and reported that neutrophils, natural killer cells, monocytes, T cells, and B cells occupied more than half of the cellular fraction when combined at 7‐ and 14‐days after VML injury. In correlation with the bulk RNA‐seq study, the pathway analysis focused on significant changes in gene sets related to the extracellular matrix and inflammation (Larouche et al., 2022). These two transcriptomic studies advanced understanding of the molecular biology changes occurring after a VML injury; however, the analysis was dominated by the robust and persistent responses of stress, inflammation, and fibrosis of this injury. A lingering question addressed in this retrospective analysis is whether there are dynamic transcriptome changes in other pathways (i.e., mitochondria and metabolic) that may influence the regenerative and reparative capacities of VML‐injured muscle?

Mitochondria play a critical role in the metabolic status of the skeletal muscle and largely maintain the free energy for ATP hydrolysis (Gibbs free energy [ΔG_ATP_]) via oxidative phosphorylation. The deleterious effect of VML injury on skeletal muscle and whole‐body metabolism has been well‐documented. For example, following VML injury, there is evidence of impaired mitochondrial respiration, reduced mitochondrial enzyme activities, and downregulated PGC1α gene and protein expression (Heo et al., 2023; McFaline‐Figueroa et al., 2023; Southern et al., 2019). Likewise, VML injury impairs whole‐body metabolism, resulting in lower oxidation of carbohydrates during diurnal hours (Raymond‐Pope et al., 2023). Despite these known physiological changes in metabolism and mitochondrial function, the extent to which VML injury alters the mitochondrial transcriptome is unclear. Therefore, the primary objective of this retrospective study is to leverage existing RNA‐seq datasets to investigate mitochondria and metabolic transcriptome changes after VML injury. This approach strongly aligns with the U.S. National Institutes of Health's strategic plan for data science to enact cost‐effective strategies to add value to existing and past research investments (NIH, 2018). Based on strong physiological data to support mitochondrial dysfunction, our central hypothesis was that the transcriptome changes to metabolic genes and pathways would be dynamic (i.e., change over time) and downregulated. Herein, we conduct a sequential analysis of the existing bulk RNA‐seq dataset (Aguilar et al., 2018) and then the single‐cell RNA‐seq dataset (Larouche et al., 2022) relying on established metabolic pathways and genes from MitoCarta3.0 to understand the mitochondria and metabolic transcriptome changes after VML injury.

## 2 METHODS

### 2.1 Experimental design

This study is a retrospective analysis, meaning that the research questions were generated after data collection. Publicly available transcriptomic datasets were identified through the Gene Expression Omnibus searching for studies that involved volumetric muscle loss injury, uninjured controls, and a time course component. Two studies were identified: (1) “Multiscale analysis of a regenerative therapy for treatment of volumetric muscle loss injury” (Aguilar et al., 2018) (GSE114799), a bulk RNA sequencing dataset and (2) “Neutrophil and natural killer cell imbalances prevent muscle stem cell–mediated regeneration following murine volumetric muscle loss” (Larouche et al., 2022) (GSE163376), a single‐cell RNA sequencing dataset. Original data files were downloaded, processed, and accessed for quality, and then analyzed.

### 2.2 Summary of study designs for original publications

**FIGURE 1:**
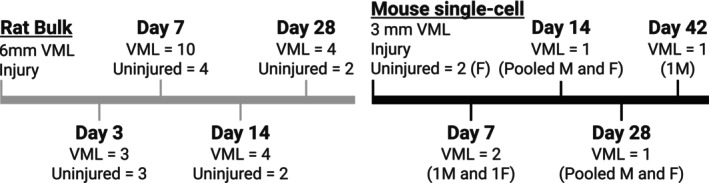
Summary of published study design time points, uninjured controls, and the number of experimental and control animals used in each retrospective study utilized for the analysis. All animals used in the Rat bulk RNA sequencing study were males.

The bulk RNA sequencing study (Aguilar et al., 2018) used an adult (~4 months of age) male Lewis rat VML injury model to the tibialis anterior muscle. The VML injury has been validated across the literature (Pollot & Corona, 2016; Wu et al., 2012). The injury removes a full thickness 6 mm punch (~86 mg; ~20% of muscle volume) to the middle third of the tibialis anterior muscle. The validation of the functional loss was tested in vivo, and the anterior compartment lost ~88%, 69%, 67%, and 59% of strength was lost at 3‐, 7‐, 14‐, and 28‐days post‐VML injury compared to control. Terminally, tissue was harvested for RNA sequencing at 3‐, 7‐, 14‐, and 28‐dpi (*n* = 3, 10, 4, and 4, respectively). The muscle remaining after injury was used for RNA isolation, and bulk RNA sequencing was completed on a Hi‐Seq 2500. Each time point included muscle samples from completely uninjured and unoperated control rats (*n* = 3, 4, 2, and 2 respectively) (Figure 1).

The single‐cell sequencing study (Larouche et al., 2022) used an adult (3–4 months of age) C57BL6/J mouse 3 mm biopsy VML injury model to the rectus femoris muscle, also a validated model across the literature (Anderson et al., 2019; Li et al., 2014) 2. This study used a combination of male and female mice but was not powered to run statistical analysis for sex differences as many of the time points reflect either a single sex (male or female) or used pooled male and female samples. Tissue was harvested for RNA sequencing at 7‐, 14‐, 28‐, and 42‐dpi. There was a single cohort of completely uninjured mice used as controls for RNA sequencing (Figure 1). The sex breakdown for each cohort was published as follows: uninjured (*n* = 2 female); 7‐dpi (*n* = 1 male, *n* = 1 female); 14‐dpi (*n* = 1 pooled sample from an undisclosed number of male and female mice); 28‐dpi (*n* = 1 pooled sample from an undisclosed number of male and female mice); and 42‐dpi (*n* = 1 male). The original published study did not report the volume of tissue removed by the 3 mm biopsy to the rectus femoris muscle; however, the injury model was validated by the functional loss of in vivo muscle torque at 28‐dpi (−62%) compared to uninjured. RNA was extracted from single cell populations after the rectus femoris muscle was dissociated and cell populations sorted by fluorescence‐activated cell sorting. 10,000–16,000 cells were sequenced on a NovaSeq 6000 using a 10× Genomics chromium single‐cell controller.

### 2.3 Data download, processing, and quality control

Rat bulk RNA sequencing data (GSE114799) (Aguilar et al., 2018) pair‐ended reads were downloaded using the SRA‐toolkit with default settings (v3.10 for Windows) (GitHub, 2025). Bulk sequencing reads were pseudo aligned to mRatBN7.2 (Genome Reference Consortium Rat build 7.2, 2021) (Howe et al., 2021) and gene counts were generated by Kallisto (v0.51.1) (Bray et al., 2016) with default settings. Reads were trimmed using Trimmomatic (v0.39) (Bolger et al., 2014) with default settings. Quality assessment was done utilizing FastQC (v0.12.1 for Windows) (Andrews, n.d.), samples that contained less than 30 million total reads or were dominated by ribosomal RNA were removed from further analysis [SRR‐7207432, ‐7207434, and ‐7207452].

Mouse single‐cell RNA sequencing data (GSE163376) (Larouche et al., 2022) was downloaded directly from the Gene Expression Omnibus database. Utilizing the Seurat package (v5.10) (Hao et al., 2024), counts data were separated by cell type and analyzed as described below. Cell subpopulations that were originally annotated as distinct subtypes (e.g., endothelial, vein, neural, progenitor, FAP, stem) were collapsed into broader categories (e.g., endothelial, neural, and FAP) to facilitate downstream analyses. Details on data processing, cell type annotations, and quality control can be found in the original publication (Larouche et al., 2022).

### 2.4 Analysis

Mitochondrial genes and gene sets were identified by utilizing the Broad Institute's MitoCarta3.0 (Rath et al., 2021). Differential gene expression analysis was performed on both datasets using the R/Bioconductor package DESeq2 (v1.44) (Love et al., 2014) by both group and time separately. Statistical significance was determined by |log_2_Fold‐Change| >1 and a false discovery rate (a Benjamini‐Hochberg adjusted *p* value) less than 0.05 (Stephens, 2016). Gene ontology data was generated using the clusterprofiler package (v4.12.0) (Wu et al., 2021). Statistically significant differentially expressed genes (DEGs) that were identified as mitochondrial were passed through the “enrichGO” function for biological processes with default settings. Gene set enrichment analysis was accomplished utilizing the R/Bioconductor package fgsea (v1.30.0) (Korotkevich et al., 2016). Following differential gene expression analysis, genes were first pre‐ranked by the Wald statistic and subsequently passed through the “fgseaMultilevel” function with default settings, a minimum gene set size of 3, and only gene sets from MitoCarta3.0 (Rath et al., 2021).

### 2.5 Data visualization and statistical analysis

**FIGURE 2:**
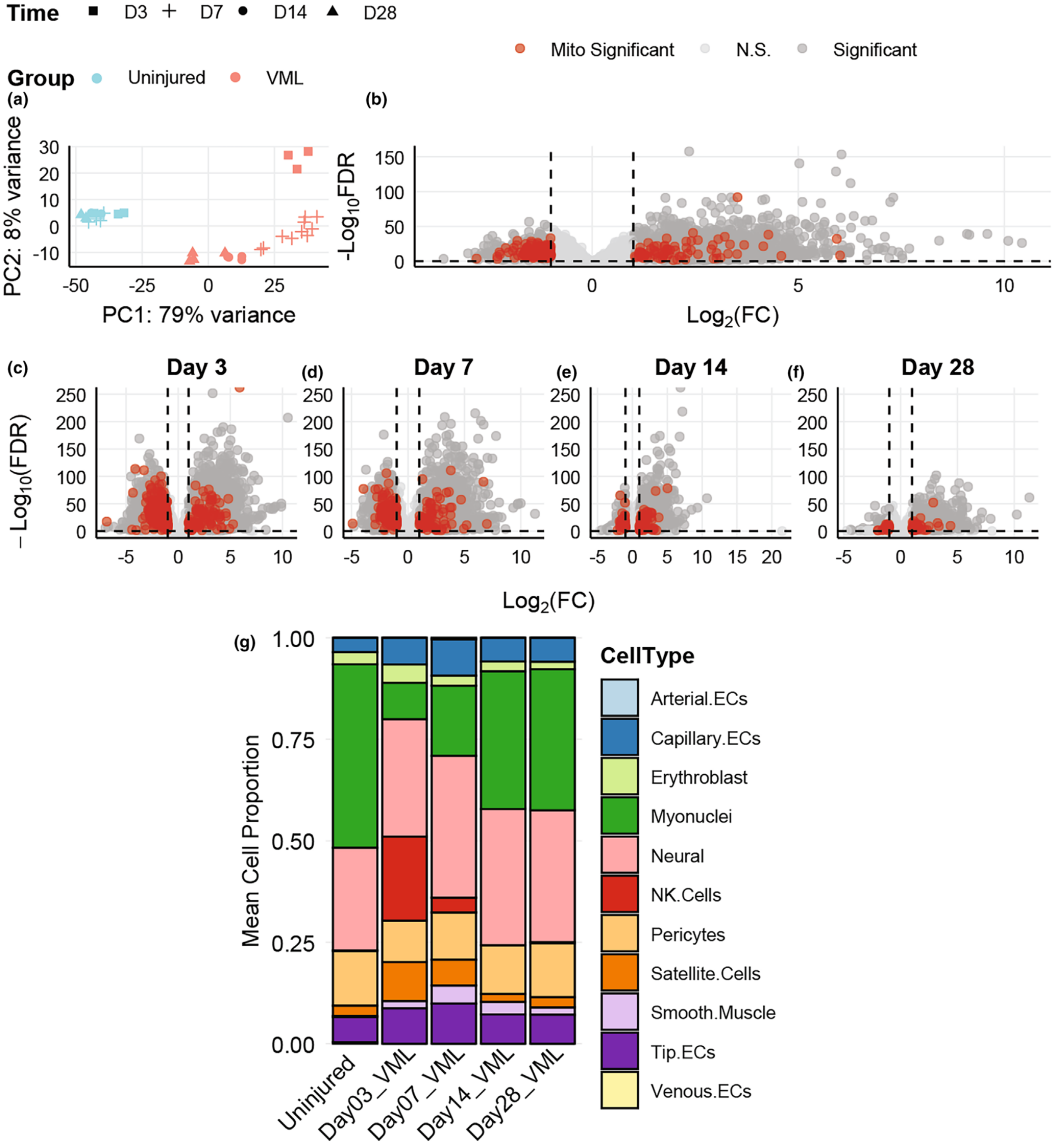
Rat sample clustering, differential gene expression analysis, and estimated mean cell proportions. (a) Principal component analysis following differential gene expression analysis of the rat bulk RNA sequencing dataset by group, time, and interaction. (b) Volcano plot of differentially expressed genes in VML group compared to uninjured control across all timepoints. (c–f) Volcano plots of differentially expressed genes at each respective day post‐VML injury. Dark gray denotes statistically significant genes, red dots denote statistically significant mitochondrial genes, and light gray indicates no statistical significance. The dotted lines indicate the cutoffs for statistical significance (false‐discovery rate (FDR) <0.05 and |log2(Fold‐Change)| >1). (g) Approximate mean cell proportions calculated by CIBERSORTx. Data reflect Uninjured (*n* = 11) and Injured (*n* = 3 at 3‐dpi, *n* = 10 at 7‐dpi, *n* = 4 at 14‐dpi, and *n* = 4 at 28‐dpi) samples from male rats. ECs denotes endothelial cells. FDR is a Benjamini–Hochberg corrected *p* value for multiple testing.

**FIGURE 3:**
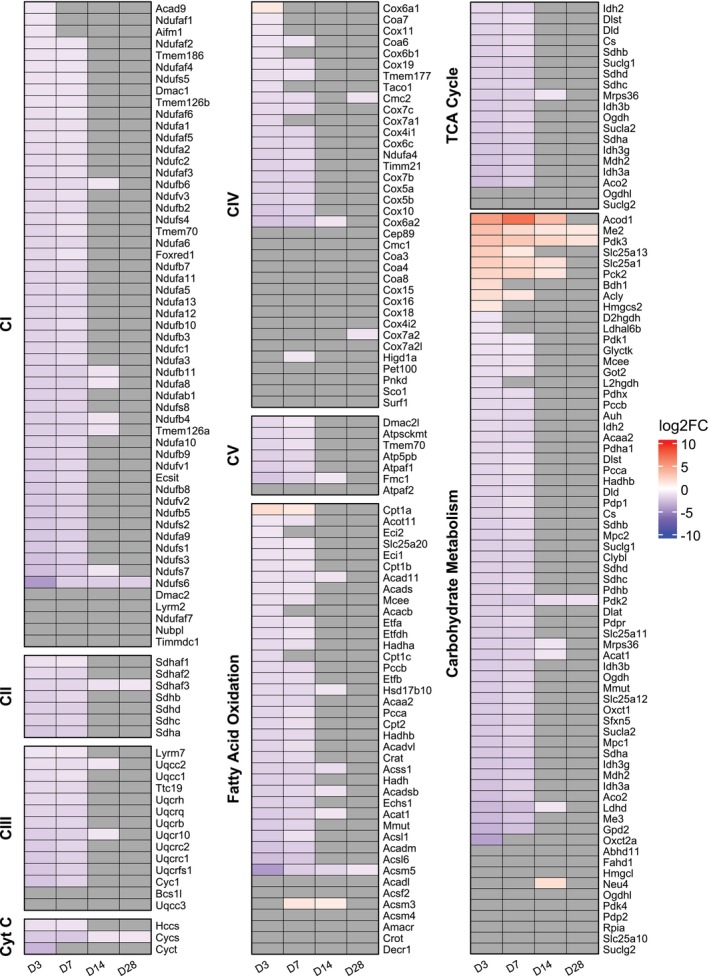
Rat heatmaps of metabolic and electron transport system gene sets. CI‐V represents complex I–V of the electron transport system. Color represents the log2Fold‐Change of each gene. Gray cells indicate genes that were not statistically significant (|log2Fold‐Change| >1 and FDR <0.05). Data reflect Uninjured (*n* = 11) and Injured (*n* = 3 at 3‐dpi, *n* = 10 at 7‐dpi, *n* = 4 at 14‐dpi, and *n* = 4 at 28‐dpi) samples from male rats.

Principal component analysis (PCA) plots were generated following differential gene expression analysis and utilizing functions of DESeq2 (Love et al., 2014), examining the full model of group, time, and interactions. Figure 2b was generated by running differential gene expression analysis by experimental group only—all VML samples against all uninjured samples. Volcano plots in Figure 2c–f were generated by comparing all uninjured samples against VML samples from each respective time point separately. Heatmaps in Figure 3 were generated by utilizing differential gene expression data (as described for Figures 2c–f) and generated using the ComplexHeatmap package (v2.20.0) (Gu et al., 2016) and circlize (v0.4.16) (Gu, 2013). Volcano plots in Figure 5 were created by running differential gene expression analysis comparing the uninjured controls against “early” (Days 7 and 14) and “late” (Days 28 and 42) VML samples with default settings. Statistical significance was determined using the same cutoffs as described above. Volcano plots of GSEA data were made following differential gene expression analysis and gene set enrichment analysis as described above. All PCA and volcano plots were generated using ggplot2 (v3.5.1) (Wickham, 2016) and ggrepel (v9.6.0) (Slowikowski, 2016). Individual panels were combined into figures utilizing package gridExtra (v2.3) (Auguie & Antonov, 2017).

Mean cell proportions were calculated using CIBERSORTx (Newman et al., 2019). The signature matrix file was generated using a scRNAseq dataset originating from the tibialis anterior muscle of a 12‐month‐old wild‐type Sprague–Dawley rat (BioSample: SAMN28901744, GEO: GSM6215661) (Taglietti et al., 2023). This single sample was processed using several filters: nFeature_RNA >200, nFeature_RNA <6000, percentage of mitochondrial reads <10%, and only genes that were expressed in at least 5 cells were retained. Data was log normalized with a scale factor of 10,000. Cell types were identified using Seurat's FindAllMarkers in conjunction with the PanglaoDB (panglaodb.se) (Franzén et al., 2019). Cell fractions were imputed using this series matrix file and the rat bulk dataset (Aguilar et al., 2018) with B‐mode batch correction, disabled quantile normalization, and 500 permutations. Individual samples were averaged within each group and reported below. Results for each sample are reported in Table S3.

To infer transcription factor activity, Virtual Inference of Protein‐activity by Enriched Regulon (VIPER) analysis was done using the viper (v1.42.0) (Alvarez et al., 2016) and dorothea (v1.20.0) (Badia‐i‐Mompel et al., 2022; Garcia‐Alonso et al., 2019; Müller‐Dott et al., 2023) packages. Results from DESeq2 were transformed using variance stabilizing transformation. The regulon was generated using only high‐likelihood interactions (“A”, “B”, and “C”). VML samples were compared against uninjured samples using a two‐sided *t*‐test. Results are reported in Tables S6 and S10.

## 3 RESULTS

To examine the variance in the rat bulk RNA sequencing dataset, a PCA was done following differential gene expression analysis. PCA of the rat dataset showed that ~79% of variance was due to the first principal component (PC1) explained by the VML injury, that is, VML versus uninjured controls (Figure 2a). The uninjured control samples cluster together tightly regardless of timepoint, while the VML‐injured samples resolve temporally (i.e., 3‐, 7‐, 14‐, and 28‐dpi), accounting for the ~8% variance shown in PC2 (Figure 2a).

Differential gene expression analysis was next done in two different ways. First, by VML injury, independent of timepoint (i.e., VML versus uninjured control) (Figure 2b, Table S1) as VML injury played the largest role in the variance of this dataset. Second, by each respective day post‐VML injury against uninjured controls (Figure 2c–f, Table S2) because the primary objective was to uncover temporal transcriptome changes after injury. The volcano plots help visualize that there are a higher number of mitochondrial‐DEGs early (3‐ and 7‐dpi) after VML injury compared to later timepoints (14‐ and 28‐dpi), and a greater percentage of those DEGs are downregulated at the early timepoints.

Relative to uninjured controls, the number of mitochondrial‐DEGs changes across time. Of the 16,912 genes analyzed in this dataset, roughly 6% (1015 genes) were identified as mitochondrial genes. The upregulated mitochondrial‐DEGs occupy roughly the same percentage of all statistically significant upregulated DEGs at each timepoint (2.3%, 1.9%, 1.9%, and 2.1% at 3‐, 7‐, 14‐, and 28‐dpi, respectively) (Table S2). In contrast, downregulated mitochondrial‐DEGs start off occupying a higher percentage of all statistically significant downregulated DEGs and drop down at Day 14 (17.9%, 20.7%, 10.4%, and 7.3% at 3‐, 7‐, 14‐, and 28‐dpi, respectively) (Table S2). This suggests a changing role in the regulation of mitochondrial genes through the first month post‐VML injury with an emphasis at 14‐dpi.

To contextualize the transcriptional shift over time, relative proportions of cell types present in the bulk dataset were calculated using both CIBERSORTx and a single‐cell reference, originating from an uninjured rat's tibialis anterior muscle (Taglietti et al., 2023). This single‐cell reference uses the same species and muscle group used in the bulk dataset. Each sample's cell proportions were calculated separately and then averaged (Table S3). Throughout the time course of repair post‐injury, there were dynamic changes in cell type composition. Most notably, the immediate appearance and subsequent disappearance of NK cells by 14 dpi (Figure 2g). As expected, there was a distinct increase in the satellite cell population that returned to baseline by 14 dpi. The proportion of myonuclei at 3 dpi was drastically less than the proportion of myonuclei in uninjured controls (9% vs. 45%, Figure 2g). The proportion of myonuclei increased by 14 dpi (34%) but did not return to levels consistent with the proportion in uninjured controls (35% by 28 dpi), a finding that is consistent with chronic deficits in muscle strength and total fiber numbers in VML‐injured muscle (McFaline‐Figueroa et al., 2025).

To understand the biological processes that the mitochondrial‐DEGs are contributing to, and to better resolve the changing transcriptome, injury‐induced mitochondrial DEGs (significantly upregulated or downregulated by ≥2‐fold) were organized into groups based on temporal profiles. Four groups were identified and include: “All Up/Down” (genes that exhibited a rapid and persistent alteration in expression), “Early Up/Down” (genes with alterations in expression overlapping 3‐ and 7‐dpi), “Middle Up/Down” (genes with alterations in expression overlapping 7‐ and 14‐day post‐injury), and “Late Up/Down” (genes with alterations in expression overlapping 14‐ and 28‐dpi). Of the 91 upregulated mitochondrial‐DEGs at 3 dpi, 30.7% (28/91) remained significant throughout 28 dpi (Table S2). In contrast, of the 488 downregulated mitochondrial‐DEGs at 3 dpi, only 3.3% (16/488) remained significant throughout the time course.

Next, using ClusterProfiler's over‐representation analysis of biological processes, Gene Ontology (GO) terms were associated with each temporal cluster for both up‐ and downregulated genes (Table 1). This analysis provides information on what biological process the mitochondrial‐DEGs contribute to, and this analysis demonstrated some marked differences between the up‐ and down‐regulated groups. For example, significant increases in the genes associated with small molecule catabolic processes and organic acid/carboxylic acid biosynthesis pathways were found in the “All Up” group, whereas the “All Down” group was dominated by decreases in genes encoding proteins associated with mitochondrial transport, mitochondrial function, and mitochondrial substrate utilization (Table 1). Interestingly, genes associated with mitochondrial transport were highly represented across several up‐ and downregulated temporal clusters, including both the “All Up” and “All Down”, “Late Up” and “Late Down”, and “Early Up” and “Middle Up” groups, suggesting an intricate modulation in expression not fully captured by this analysis (Table 1). Genes associated with GO terms “oxidative phosphorylation,” “aerobic respiration,” “cellular respiration,” “ATP synthesis coupled electron transport,” “respiratory electron transport system,” “electron transport system,” “aerobic electron transport system,” and “mitochondrial respiratory chain complex assembly” were exclusively associated with downregulated gene temporal groups (Table 1). Also, “fatty acid metabolic process” was associated with all four downregulated temporal groups (Table 1).

**TABLE 1:** Rat over‐representation analysis of Gene Ontology (GO) terms for temporal groups categorized by up‐ or down‐regulated.

Temporal group	Upregulated	Downregulated
All	Small molecule catabolic process (25%) [6/24]	Mitochondrial transport (27%) [4/15]
	Organic acid biosynthetic process (25%) [6/24]	Cellular respiration (27%) [4/15]
	Carboxylic acid biosynthetic process (25%) [6/24]	Energy derivation by oxidation of organic compounds (27%) [4/15]
	Mitochondrial transport (21%) [5/24]	Generation of precursor metabolites and energy (27%) [4/15]
		Import into the mitochondrion (20%) [3/15]
		Mitochondrial transmembrane transport (20%) [3/15]
		Dicarboxylic acid metabolic process (20%) [3/15]
		Oxidative phosphorylation (20%) [3/15]
		Aerobic respiration (20%) [3/15]
		Positive regulation proteolysis (20%) [3/15]
		Purine ribonucleotide metabolic process (20%) [3/15]
		Ribonucleotide metabolic process (20%) [3/15]
		Fatty acid metabolic process (20%) [3/15]
		Regulation of proteolysis (20%) [3/15]
		Ribose phosphate metabolic process (20%) [3/15]
Early	Mitochondrial transport (21%) [13/63]	Generation of precursor metabolites and energy (25%) [94/372]
		Energy derivation by oxidation of organic compounds (23%) [85/372]
		Cellular respiration (23%) [85/372]
		Aerobic respiration (20%) [74/372]
Middle	Small molecule catabolic process (20%) [9/43]	Generation of precursor metabolites and energy (29%) [16/55]
		Cellular respiration (25%) [14/55]
		Energy derivation by oxidation of organic compounds (25%) [14/55]
		Small molecule catabolic process (25%) [14/55]
		Oxidative phosphorylation (24%) [13/55]
		Aerobic respiration (24%) [13/55]
		Organic acid catabolic process (24%) [13/55]
		Carboxylic acid catabolic process (24%) [13/55]
		Fatty acid metabolic process (24%) [13/55]
		Electron transport system (20%) [11/55]
Late	Small molecule catabolic process (25%) [7/28]	Dicarboxylic acid metabolic process (25%) [4/16]
	Mitochondrial transport (21%) [6/28]	Mitochondrial transport (25%) [4/16]
	Carboxylic acid biosynthetic process (21%) [6/28]	Cellular respiration (25%) [4/16]
	Organic acid biosynthetic process (21%) [6/28]	Energy derivation by oxidation of organic compounds (25%) [4/16]
		Generation of precursor metabolites and energy (25%) [4/16]

*Note*: Percentage of the genes in each group that are associated with a given GO term are in parentheses and calculated as [Number of genes associated with a GO term/Total genes in each group as identified by Gene Ontology]. Note that not all genes were able to be identified by GO.

The Gene Ontology biological process analysis largely supported physiological data indicating a decline in VML‐injured muscle fiber mitochondrial function. To support the central hypothesis that the mitochondrial transcriptome is changing in correlation with established physiological deficits, we arranged individual genes into their respective associations with the electron transport system, Krebs cycle, fatty‐acid oxidation, and carbohydrate metabolism (Figure 3). Genes associated with protein subunits of the electron transport system show a robust downregulation early (3‐ and 7‐dpi) that mostly resolves by 14‐dpi (Figure 3 CI–CV, and Cytochrome C). Mitochondrial complexes I, II, III, and V each had 85% or greater downregulated genes as a percentage of total genes and had no genes upregulated at any individual timepoint post‐VML injury (Figure 3 CI, CII, CIII, CV, and Cytochrome C).

The electrons shuttling across the electron transport system are removed from carbon fuel sources by redox reactions in critical metabolic processes within carbohydrate and lipid metabolism and the TCA cycle (Fisher‐Wellman et al., 2018). Visualization using a heatmap indicates trends for the electron transport system; genes associated with proteins of the Krebs cycle, fatty‐acid oxidation, and carbohydrate metabolism show a robust downregulation at 3 dpi that mostly resolves by 14 dpi, and each heatmap showed differential gene regulation of 85% or greater of the total respective gene pool (Figure 3 TCA Cycle, Fatty Acid Metabolism, and Carbohydrate Metabolism). In contrast to the heatmaps for the electron transport system, there is evidence of upregulated DEGs, particularly within genes associated with proteins for carbohydrate metabolism (Figure 3 Carbohydrate Metabolism).

One DEG to highlight is *Pdk3* that was upregulated across all timepoints. *Pdk3* encodes for pyruvate dehydrogenase kinase that inhibits pyruvate dehydrogenase activity, and a pyruvate dehydrogenase activity bottleneck in carbohydrate metabolism is reported in VML‐injured muscle fibers (Heo et al., 2023). Only 6 of the mitochondrial DEGs associated with the electron transport system, Krebs cycle, fatty‐acid oxidation, or carbohydrate metabolism were significant across all timepoints (*Acsm5*, *Cycs*, *Me2*, *Pdk2*, *Pdk3*, *Ndufs6*, and *Sdha3*). Interestingly, *Pdk2* also encodes for a pyruvate dehydrogenase kinase isoform, yet was significantly downregulated across all time points in contrast to *Pdk3*. *Me2* also encodes for a pyruvate‐associated protein and was also upregulated across all timepoints. Specifically, *Me2* encodes for mitochondrial NAD‐dependent malic enzyme that participates in pyruvate recycling in a reduced glycolysis environment (Hsieh et al., 2023; Hsu & Lardy, 1967; Loeber et al., 1991).

**FIGURE 4:**
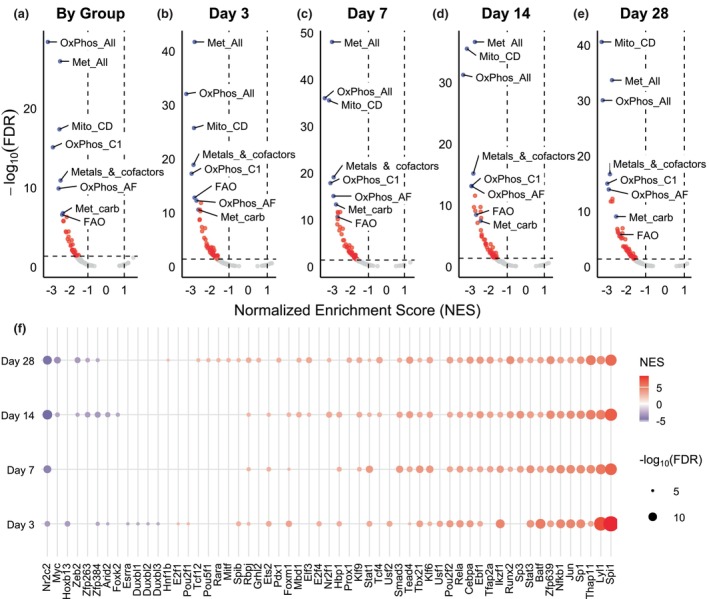
In the Rat dataset, oxidative phosphorylation (OxPhos_All), metabolism (Met_All), and mitochondrial central dogma (Mito_CD) are the leading mitochondrial gene sets across all time points. (a–e) Volcano plots of pre‐ranked (Wald statistic) GSEA by experimental group and time point. Red dots denote significant mitochondrial gene sets. Gray dots denote nonsignificant gene sets. Blue dots denote annotated gene sets. The dotted lines indicate the cutoffs for statistical significance (|NES| >1 and FDR <0.05). (f) Dot plot of significant transcription factors by VIPER analysis. Dot size denotes −log10(FDR) and color indicates NES. Significant transcription factors are (|NES| >2.5 and FDR <0.05). Data reflect Uninjured (*n* = 11) and Injured (*n* = 3 at 3‐dpi, *n* = 10 at 7‐dpi, *n* = 4 at 14‐dpi, and *n* = 4 at 28‐dpi) samples from male rats. AF, assembly factors; C1, complex I; carb, carbohydrate; CD, central dogma; FAO, fatty acid oxidation; Met, metabolism; Mito, mitochondrial; OxPhos, oxidative phosphorylation.

In contrast to the Gene Ontology analysis, gene set enrichment analysis (GSEA) helps elucidate what changes in the entire transcriptome were a result of VML injury. GSEA is a type of overrepresentation analysis that determines the degree to which a given set of genes is present at either extreme of a ranked gene list, returning an enrichment score that is normalized to account for differences in both gene set size and in correlations between gene sets and the expression dataset (Korotkevich et al., 2016). For statistical comparisons, the FDR, a Benjamini‐Hochberg adjusted *p* value, was applied to account for multiple testing (Stephens, 2016). All genes were pre‐ranked by the Wald statistic and passed through GSEA utilizing The Broad Institute's MitoCarta3.0, a resource for mitochondrial genes and gene sets (Rath et al., 2021). Independent of time post‐VML injury, 37 of the 119 defined mitochondrial gene sets were statistically significant for VML‐injured relative to uninjured controls (Figure 4a, Table S4); whereas, there were 60, 68, 58, and 65 significant mitochondrial gene sets at days 3‐, 7‐, 14‐, and 28‐days post VML injury, respectively (Figure 4b–e, Table S5). Despite there being both up‐ and down‐regulated individual mitochondrial DEGs (Figure 2), there were no statistically significant upregulated gene set pathways.

The eight most significantly downregulated gene sets in the VML versus uninjured control (independent of time) represent gene sets encoding for proteins associated with oxidative phosphorylation (OxPhos_All), mitochondrial central dogma (Mito_CD), metabolism (Met_All), complex I of the respiratory chain (OxPhos_C1), metabolism of metals and cofactors (Metals_&_cofactors), oxidative phosphorylation assembly factors (OxPhos_AF), carbohydrate metabolism (Met_Carb), and fatty acid oxidation (FAO) (Figure 4b–e). By 14 dpi, fatty‐acid oxidation and carbohydrate metabolism become less significant, and metabolism of mitochondrial RNA, lipid metabolism, protein import and sorting, branch‐chain amino acid metabolism, protein import sorting and homeostasis, and amino acid metabolism become more prominently downregulated (Table S5).

To better understand what transcriptional elements could be driving this dynamic landscape, a VIPER (Virtual Inference of Protein‐activity by Enriched Regulon) analysis was performed to infer transcription factor (TF) protein activity (Figure 4f, Table S6). This analysis infers the protein activity of a given TF by analyzing the transcripts of its regulon. Of the 161 TFs examined, 57 (35.4%) were significantly active at any time point. At 3‐, 7‐, 14‐, and 28‐dpi there were 36, 28, 38, and 42 significant TFs, respectively. Of the significant TFs, the majority were upregulated compared to uninjured control (77%, 96%, 82%, and 88% at 3‐, 7‐, 14‐, and 28‐dpi, respectively). Moreover, of the 20 TFs that were significant across all time points, only one was a downregulated TF.

The most significantly upregulated TF across all time points was Spi1 (also known as PU.1), an ETS‐domain TF known to be required for the development of both myeloid and lymphoid lineages (Scott et al., 1994). Of the remaining TFs significantly upregulated across all time points, 4 categories of biological processes emerged: (i) immune regulation (e.g., Tbx21, Nfkb1, and Stat3) (Hillmer et al., 2016; Liu et al., 2017; Stolarczyk et al., 2014; Woo et al., 2014), (ii) fibrosis/tissue remodeling (e.g., Tead4 and Klf6) (Benhaddou et al., 2012; Miele et al., 2008; Sawaki et al., 2015), (iii) cell proliferation/stress response (e.g., Jun, Sp1, and Thap11) (Johnson & Nakamura, 2007; Parker et al., 2012; Poché et al., 2016; Vizcaíno et al., 2015), and (iv) development/cell fate control (e.g., Rbpj, Pou2f2, and Lyl1) (Capron et al., 2006; Javed et al., 2020; Tanigaki et al., 2002).

In contrast, Nr2c2 was the only TF to be significantly downregulated at all time points. Nr2c2 (also known as TR4) belongs to the nuclear hormone receptor family and is known to be involved in many biological processes such as development, cellular differentiation, and homeostasis (Lee et al., 2011; Liu et al., 2025; Shyr et al., 2009). Interestingly, Myc, an established cell cycle regulator and more specifically a regulator of skeletal muscle development and regeneration, was not significantly active at 3‐ and 7‐dpi. However, its activity was significantly downregulated at 14‐ and 28‐dpi despite its expression being significantly upregulated at 3‐, 7‐, and 14‐dpi (Table S2). This mismatch between expression and inferred activity suggests a dysregulation in the transcriptional function of this key TF during VML injury repair.

In summary, the individual mitochondrial‐DEGs, estimated cell proportions, Gene Ontology, GSEA, and VIPER results support a cellular environment post‐VML injury in which there is a change in the regulation of the mitochondrial transcriptome, mostly in the downregulation direction. To advance and further evaluate the testing of the central hypothesis, a similar analysis of a mouse single‐cell RNA sequencing dataset was conducted.

The original reporting of the single‐cell RNA sequencing dataset from mice with VML injury separated the muscle into 16 different cell types at 7‐, 14‐, 28‐, and 42‐dpi: B cells, dendritic, endothelial, erythroblast, FAPs, lymph, macrophage, monocytes, myonuclei, neural, neutrophil, pericyte, satellite, smooth muscle, T/NK, and tenocyte cell types (Larouche et al., 2022). In contrast to the rat dataset, where roughly 6% of all genes were identified as mitochondrial, only about 4% of the 31,053 mouse genes were identified as mitochondrial. Another key difference between the rat and mouse datasets is the number of uninjured control samples included in the original study design. This mouse dataset contained only two uninjured controls total, in contrast to the rat dataset that had uninjured controls at each time point (*n* = 3, 4, 2, and 2 for days 3, 7, 14, and 28, respectively, Figure 1) (Aguilar et al., 2018; Larouche et al., 2022). A PCA of each cell type revealed time points 28‐ and 42‐dpi to be closely related to each other as well as closely related to uninjured controls for most cell types (Figure S1). This correlates in part with the rat dataset, in which there were fewer mitochondrial‐DEGs at the later time points (14‐ and 28‐dpi) compared to the early time points (3‐ and 7‐dpi) (Figure 2c–h). To overcome being underpowered for the analysis of the single‐cell RNA sequencing data and because of the PCA clustering, mitochondrial‐DEGs and GSEA were determined after combining the 7‐ and 14‐day mouse data to create an “early” temporal group and combining the 28‐ and 42‐day mouse data to create a “late” temporal group.

**FIGURE 5:**
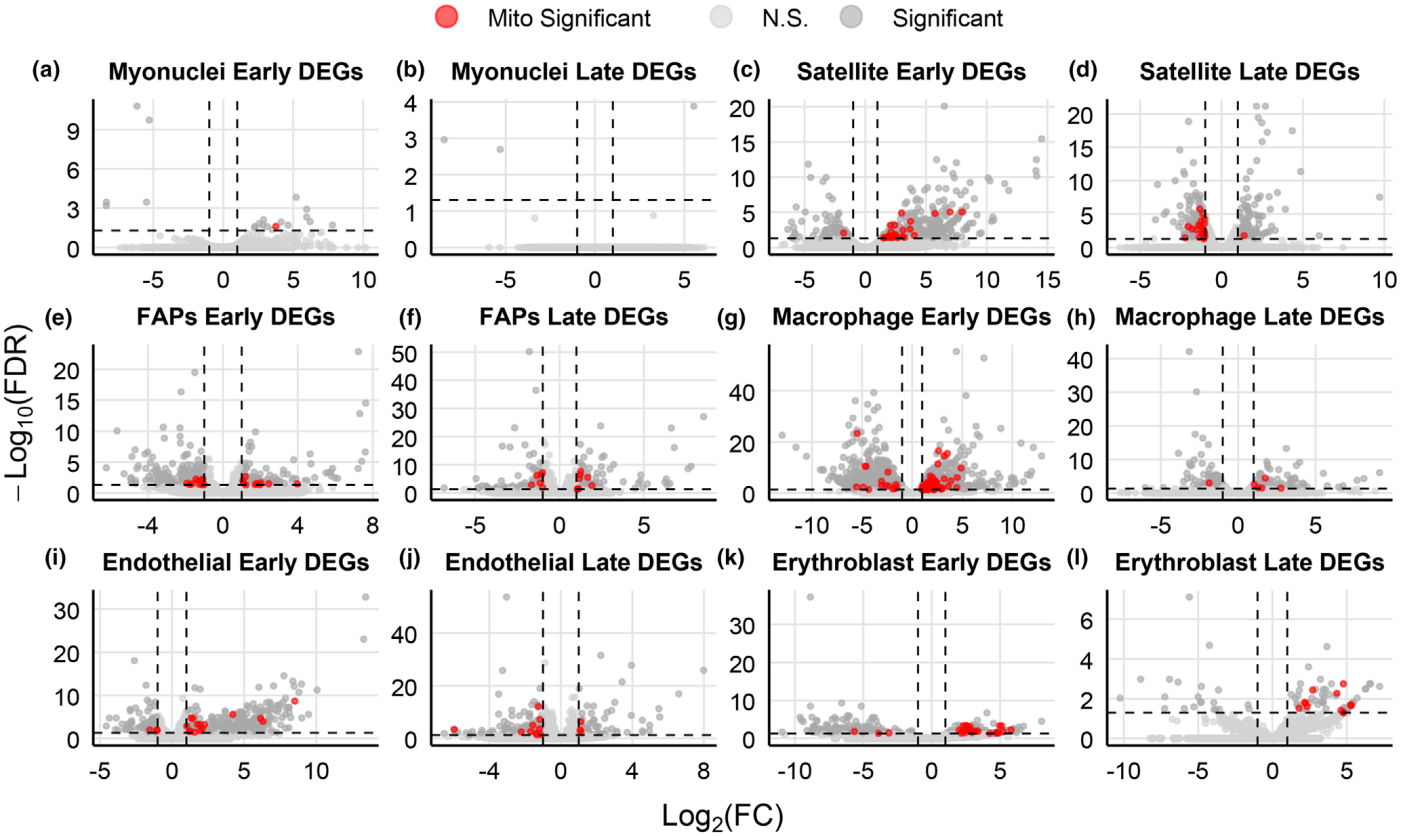
Mouse myonuclei, satellite cells, FAPs, macrophages, endothelial, and erythroblasts DEGs show unique expression patterns following VML injury. Volcano plots of differentially expressed genes in myonuclei (a, b), satellite cells (c, d), FAPs (e, f), macrophages (g, h), endothelial (i, j), and erythroblasts (k, l). Dark gray denotes statistically significant genes, red dots denote statistically significant mitochondrial genes, and light gray indicates no statistical significance. The dotted lines indicate the cutoffs for statistical significance (|log2(Fold‐Change)| >1 and false‐discovery rate (FDR) <0.05). Data reflect Uninjured (*n* = 2 female) and Injured (Early, *n* = 1 male, *n* = 1 female, *n* = 1 pooled male and female; Late, *n* = 1 pooled male and female, *n* = 1 male) samples from mice.

Differential gene expression analysis was done for early and late temporal groups, and then volcano plots were generated for a subset of six cell types (Figure 5). These specific cell types were selected based on their relevance to muscle pathology in the context of VML injury and having the greatest proportion of significant mitochondrial DEGs (Table S7). In contrast to the volcano plots for the bulk RNA sequencing rat dataset, there is no consistent pattern of changes in the proportion of mitochondrial DEGs nor upregulation or downregulation across the cell types with time post‐VML injury (Figure 5). The proportion of DEGs identified as mitochondrial for the early temporal group was myonuclei with 5.0% (1/20), satellite cells with 6.1% (27/445), FAPs with 5.9% (20/338), macrophages with 8.0% (154/1915), endothelial cells with 3.2% (18/559), and erythroblasts with 7.8% (26/334). In the late temporal group, these proportions were mostly less, as shown by myonuclei cells with 0.0%, satellite cells with 5.8% (21/365), FAPs with 5.1% (14/273), macrophages with 3.0% (6/200), endothelial cells with 4.3% (12/300), and erythroblasts with 14.0% (12/86). While all cell types saw a decrease in the number of all DEGs and mitochondrial DEGs, macrophages had the largest decrease in both number and proportion, and erythroblast had the largest change as well as being the only cell type to see an increase in the proportion of mitochondrial DEGs.

The volcano plots help visualize additional contrasts between these two datasets. Whereas there was little evidence of changing regulation (upregulated to downregulated or vice versa) over time in the rat dataset, the distribution of mitochondrial‐DEGs in satellite cells switched from predominantly upregulated in the early temporal group to downregulated in the late temporal group (Figure 5c,d). A similar trend was also seen in endothelial cells (Figure 5i,j). Other notable observations for individual cell types include that myonuclei cells had only one mitochondrial‐DEG, *Gpx1*, a glutathione peroxidase (Figure 5a,b) (all cell type mitochondrial DEGs are reported in Table S8) and macrophages had much fewer mitochondrial‐DEGs in the late temporal group compared to the early temporal group, representing a 5% decrease in the proportion of mitochondrial‐DEGs.

**FIGURE 6:**
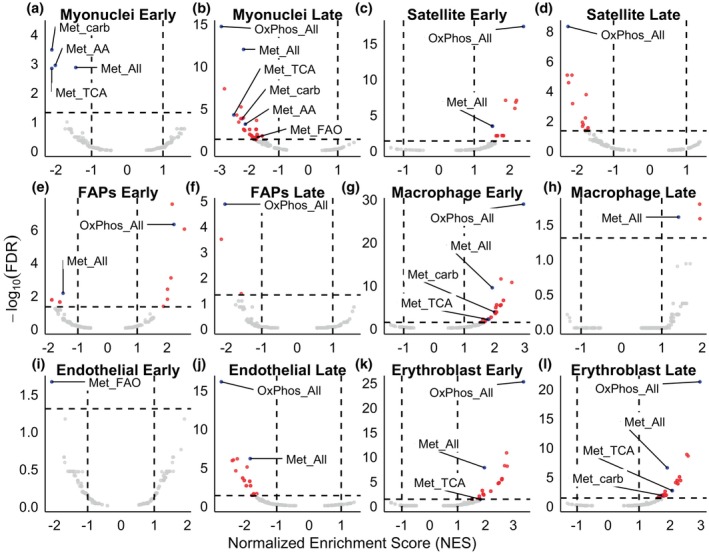
Mouse GSEA Results in myonuclei (a, b), satellite (c, d), FAPs (e, f), macrophage (g, h), endothelial (i, j), and erythroblasts (k, l). Red dots denote statistically significant mitochondrial gene sets, blue denotes annotated significant gene sets, and light gray indicates no statistical significance. The dotted lines indicate the cutoffs for statistical significance (false‐discovery rate (FDR) <0.05 and |log2(Fold‐Change)| >1). Data reflect Uninjured (*n* = 2 female) and Injured (Early, *n* = 1 male, *n* = 1 female, *n* = 1 pooled male and female; Late, *n* = 1 pooled male and female, *n* = 1 male) samples from mice. Met is short for metabolism, carb is short for carbohydrate, and AA stands for amino acid.

The advantage of utilizing a GSEA to further interrogate the changing mitochondrial transcriptome was exemplified through the myonuclei cells. Despite having only one mitochondrial DEG (Figure 5a,b), myonuclei cells had 4 significant mitochondrial gene sets in the early temporal group and 29 significant mitochondrial gene sets in the late temporal group (Figure 6a,b). Notably, all the significant mitochondrial gene sets were downregulated in myonuclei. The satellite cell GSEA revealed a pattern of regulation similar to that of the satellite cell DEG volcano plots (Figure 5c,d) in that the early temporal group had upregulated mitochondrial gene sets and the late temporal group had downregulated mitochondrial gene sets (Figure 6c,d). Also, consistent with the individual DEG analysis, macrophages displayed a high regulation of mitochondrial gene sets early (*n* = 23, Figure 6g) relative to late (*n* = 3, Figure 6h).

Finally, cells with high proliferation attributes early after skeletal muscle injury (e.g., satellite cells, FAPs, and macrophages) showed a similar mitochondrial gene set regulation. Specifically, there were greater upregulated mitochondrial gene sets in the early temporal group and greater downregulated mitochondrial gene sets in the late temporal group. Notably, genes associated with oxidative phosphorylation (OxPhos_All) were among those that flipped from being upregulated to downregulated (in satellite cells and FAPs, Figure 6c–f) or from being upregulated to not significantly regulated (in macrophages, Figure 6g,h). This may reflect the changing energetic demands of proliferation in these cell types. The entire GSEA for all 16 cell types is reported in Table S9.

**FIGURE 7:**
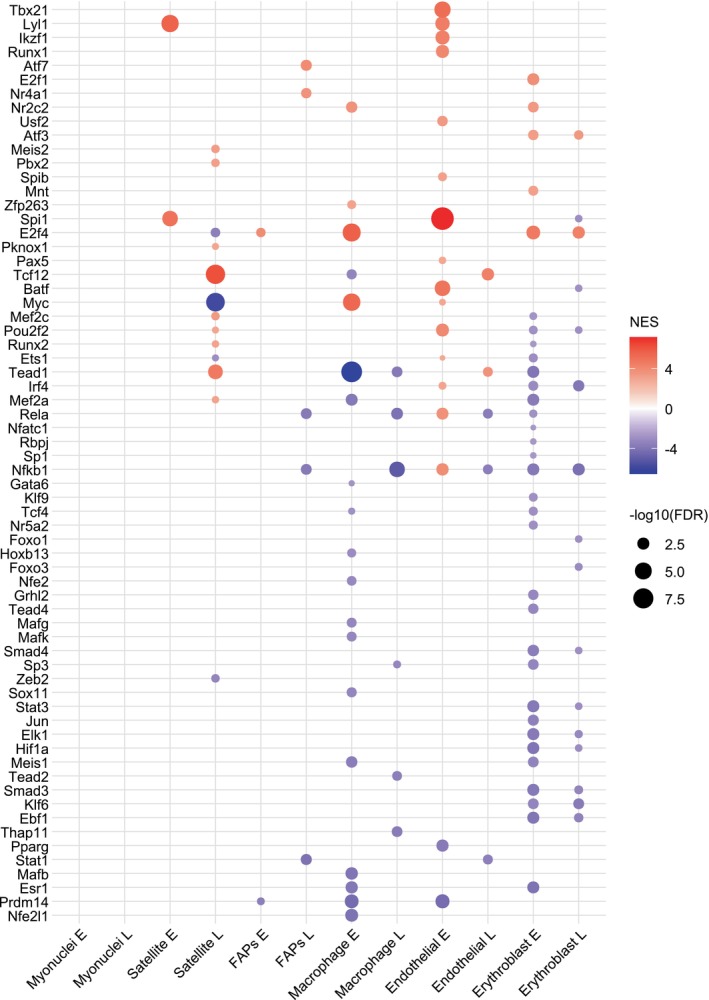
Mouse dot plot of significant transcription factors by VIPER analysis. Dot size indicates FDR and color indicates NES. Significant transcription factors are (|NES| >2.5 and FDR <0.05). Data reflect Uninjured (*n* = 2 female) and Injured (Early, *n* = 1 male, *n* = 1 female, *n* = 1 pooled male and female; Late, *n* = 1 pooled male and female, *n* = 1 male) samples from mice. Early temporal groups are indicated by the letter E and the late temporal groups are indicated by the letter L.

As described previously, VIPER analysis revealed 94 statistically significant TFs in the single‐cell dataset (Figure 7, Table S10). Out of the 170 available TFs, 55.3% were significant, which is higher than the bulk dataset's 35.4% (57/161 TFs). Of the 32 groups (16 cell types, early and late temporal groups), 14 groups were comprised mostly of upregulated TFs. Conversely, 10 were comprised mostly of downregulated TFs, two were evenly split, and the last six had no significant TFs. Erythroblasts in the early temporal group had the most significant TFs with 33. Notably, myonuclei and neutrophils were the only cell types to not have a significant TF, early or late. The two most significantly upregulated TFs from the bulk dataset, Spi1 and Lyl1, were significantly upregulated in dendritic early and late temporal groups, T/NK cells early and late temporal groups, satellite early, and endothelial early temporal group. Interestingly, Nr2c2, the most downregulated TF in the bulk dataset, was not downregulated here but significantly upregulated in seven groups including monocyte early and late temporal groups, as well as in the early temporal groups of macrophage, dendritic, B cell, erythroblast, and smooth muscle cell types. Interestingly, Myc, the other noted downregulated TF in the bulk dataset, was significantly downregulated in satellite, smooth muscle, and pericyte cell type in the late temporal group. While in early temporal groups, Myc was significantly upregulated in five cell types: dendritic, endothelial, lymph, macrophage, and monocyte. The entire VIPER analysis and extra details are reported in Table S10.

In summary, the individual mitochondrial‐DEGs, GSEA, and VIPER results highlight the unique cellular responses during the first 42 days post‐VML injury as well as their similarities. While each cell type was unique in its transcriptional response, cell types with high proliferative attributes showed similar trends in gene expression. Furthermore, cell types within the hemopoietic lineage also showed similar transcriptional trends but were not uniform across all cell types or temporal groups.

## 4 DISCUSSION

The primary objective of this retrospective analysis was to investigate the temporal regulation of metabolic transcriptomes after VML injury. There are three primary conclusions based upon the results. First, bulk RNA sequencing dataset analysis strongly supports a downregulation of metabolic transcriptomes consistent with established physiological evidence of mitochondrial dysfunction in the remaining muscle after injury. Second, the single‐cell sequencing dataset analysis highlights cell‐type‐specific patterns of metabolic transcriptome regulation that can be leveraged to better target tissue repair and regenerative mechanisms post‐VML injury. Third, temporal analysis of the bulk and single‐cell datasets reveal a pattern of metabolic transcriptome regulation that correlates with VML pathology stabilizing around 14‐dpi.

An inherent limitation of a retrospective analysis is that it relies on the study design of the previously peer‐reviewed selected studies. While the strengths of the two published studies were used in building the rationale for this retrospective study (e.g., time course analysis and uninjured controls), there are important considerations that include: a single sex (male) used in the bulk RNA sequencing study; and small animal numbers and pooled male/female samples from the single‐cell sequencing study. Another notable limitation is the reference dataset (Taglietti et al., 2023) used to generate approximate cell proportions in the bulk dataset, which lacked cell‐type diversity seen in the single‐cell dataset (e.g., macrophages, FAPs, and neutrophils).

### 4.1 Regulation of metabolic transcriptome supports mitochondrial dysfunction

There was robust evidence in support of the central hypothesis that the mitochondrial transcriptome was downregulated following VML injury in support of established mitochondrial dysfunction. Mitochondrial dysfunction following VML injury is marked by lower oxygen consumption and electron conductance rates in the presence of either carbohydrate or lipid carbon fuel sources (Heo et al., 2023; McFaline‐Figueroa et al., 2022, 2023). Electron conductance can be affected by the function of dehydrogenases within glycolysis, Krebs cycle, and fatty‐acid oxidation [e.g., pyruvate dehydrogenase (PDH), succinate dehydrogenase (SDH), and hydroxyacyl coenzyme A dehydrogenase (BHAD)] and by the function of the electron transport system complexes (i.e., complexes I–IV). Electrons are removed from carbon‐based fuel sources by dehydrogenases within glycolysis, Krebs cycle, and fatty‐acid oxidation, then transferred to the electron transport system by electron carriers (e.g., NADH). There, electrons enter the electron transport system by redox reactions at mitochondrial complexes ‐I and ‐II. Electrons then pass through complex‐III before ultimately being received by oxygen at complex‐IV. The bulk RNA sequencing indicates that greater than 85% of the DEGs were overwhelmingly downregulated for the four mitochondrial complexes. There was also a pattern of downregulation for genes associated with dehydrogenases within the mitochondria, for example, PDH (*PDHA*, *PDHB*, *PDP1*, and *PDHX*), SDH (*SHDA*, *SDHB*, and *SDHC*), and BHAD (*HADH*) (Figure 3).

How the downregulation of the mitochondrial transcriptome manifests into physiological dysfunction after VML injury is still unclear. One might expect diminished mitochondrial enzyme activities (e.g., complex‐I activity) after VML injury, considering the downregulated transcriptomes; however, there is mixed evidence showing examples of both decreased activity (Heo et al., 2023) and no changes at all (Dalske et al., 2021; Heo et al., 2023) across several enzymes analyzed. The disconnect between transcriptome regulation and protein function could be due to mitochondrial content regulation after VML injury. The average half‐life for mitochondrial proteins ranges between 5 and 18 days depending on the tissue (Beattie et al., 1967; Krishna et al., 2021; Stauch et al., 2023). A proteomic analysis revealed a higher abundance of proteins related to oxidative phosphorylation from enriched mitochondrial fractions at 14‐dpi compared to uninjured (Heo et al., 2025), and among the mitochondrial pathways shown to be significantly downregulated via the GSEA were genes associated with mitochondrial protein removal and degradation, such as protease genes, mitochondrial fission genes, and mitochondrial autophagy genes (Table S4). Citrate synthase activity is an established marker for mitochondrial content in skeletal muscle (Larsen et al., 2012) and a consistent finding across several VML studies is that citrate synthase activity is not diminished after VML and, in some studies, is increasing over time post‐VML (Corona et al., 2018) or significantly higher than uninjured muscle (Chao et al., 2019). The implication being that there may be a slower turnover of mitochondrial proteins after VML injury that can affect the analysis of individual enzyme activities and not immediately reflect the changing mitochondrial transcriptome.

Mitochondrial function is also dependent on the mitochondrial network organization and size. For example, during times of high energy demand, a well‐connected mitochondrial network can distribute energy more effectively (Glancy et al., 2015, 2017) and is facilitated by the structure of the inner‐mitochondrial cristae and by intermitochondrial junctions. The number and size of intermitochondrial junctions varies across skeletal muscle fiber types, and changes in intermitochondrial junction number and size are thought to play a role in restricting the spread of mitochondrial network dysfunction when there is cellular stress or damage (Bleck et al., 2018). The bulk RNA sequencing GSEA revealed several downregulated pathways that contribute to the regulation of the mitochondrial network organization. These included downregulated pathways for mitochondrial fission and fusion, mitochondrial membrane interaction, and genes associated with the protein assembly and structure of the inner‐mitochondrial membrane (Table S4). These transcriptomic changes correlate with evidence of a highly disorganized mitochondrial network following VML injury (Southern et al., 2019) and encourage further investigation into targeting mitochondrial network maintenance pathways to improve the pathophysiology.

### 4.2 Unique metabolic transcriptome regulations in satellite cells, FAPs, and macrophages provide targets for future intervention

Stem cells demonstrate metabolic reprogramming to maintain pluripotency and undergo differentiation (Cliff & Dalton, 2017; Iworima et al., 2024; Meleshina et al., 2016). Stem cells commonly found in skeletal muscle include satellite cells that differentiate into new myofibers, FAPs that can differentiate into adipose tissue or fibroblasts, and monocytes that differentiate into macrophages. In the context of VML injury, there is strong evidence of a robust inflammatory response and macrophage presence within the remaining muscle tissue (Corona et al., 2018; Larouche et al., 2022), and moderate evidence for diminished satellite cell activity (Anderson et al., 2019) and dysregulated FAP activation (Hymel et al., 2023). Results from the temporal analysis of single‐cell RNA sequencing data showed an intriguing similarity for the metabolic transcriptome regulation for satellite cells, FAPs, and macrophages. Specifically, there was an early upregulation (i.e., up to 14 dpi) and then either a reversal to downregulation (i.e., for satellite cells and FAPs) or a return to baseline (i.e., macrophages). One example of a gene known to facilitate this metabolic reprogramming is hypoxia‐inducible factor 1α (*Hif1α*) (Corcoran & O'Neill, 2016). There was a significant early upregulation of *Hif1α* in macrophages and FAPs, but not satellite cells (Table S6). This point is made to highlight how our retrospective analysis could be used to identify genetic targets for addressing the pathophysiology after VML, in this case diminished satellite cell activation.

Transcription factors are critical regulatory proteins that bind DNA to promote or inhibit the expression of specific genes. Several TFs play key roles in regulating metabolism and mitochondrial function. Findings from the bulk dataset yielded few notable TFs with potential mitochondrial gene regulation evidence, that is, Tead4 and Nr2c2 (Figure 4f, Table S6). *Tead4* is known to be able to localize to both the nucleus and mitochondria, influencing gene expression and mitochondrial function (Chen et al., 2021; Hsu et al., 2022; Kaneko & DePamphilis, 2013; Kumar et al., 2018). A Tead4 knockdown study in mouse trophoblast stem cells further demonstrated this TF's ability to bind mitochondrial DNA and influence the expression of mitochondrial‐encoded genes directly involved in and related to the ETS (Kumar et al., 2018). Another Tead4 knockdown study further demonstrated how Tead4 influences nuclear‐encoded mitochondrial gene expression, and its subcellular localization was arginine dependent (Chen et al., 2021). Studies have shown Tead4 modulates mitochondrial functions such as respiration rates, reactive oxygen species generation, and membrane potential (Chen et al., 2021; Hsu et al., 2022; Kaneko & DePamphilis, 2013; Kumar et al., 2018). While Tead4 was upregulated in the bulk study, its expression, subcellular localization, and activity as a TF could be leveraged to target cell types such as endothelial cells where its activity is downregulated.

Conversely, Nr2c2 (also known as TR4), which was downregulated throughout the bulk study's injury time course, is an orphan nuclear receptor that's been shown to be involved in multiple broad cellular and physiological functions (PubChem, n.d.; Ding et al., 2013; Liu et al., 2025). A Nr2c2 knockout study resulted in mitochondrial myopathy (Liu et al., 2011). Results from this study showed serum lactate increased by a factor of 2.5 as well as impaired complex I activity and ATP production, which were nearly halved. This mitochondrial myopathy was partially explained by the decreased expression of complex I assembly factor *Ndufaf1*. Interestingly, the transcriptional activity of Nr2c2 was upregulated in multiple hematopoietic lineage cell type groups as well as smooth muscle in the early temporal group. While Nr2c2 was not differentially expressed in either dataset, its activity as a TF could be leveraged as a potential target to address the mitochondrial dysfunction of VML.

The single‐cell data showed a more nuanced picture of potential TF‐mediated mitochondrial gene regulation (Figure 7, Table S10). There are cases where a TF is upregulated in one cell type but downregulated in another cell type, such as Mef2a. Mef2a is known to be involved in myoblast proliferation and differentiation, and the homozygous knockout of Mef2c resulted in embryonically lethal (Akhtar et al., 2012; Lin et al., 1997; Naya & Olson, 1999; Wang et al., 2018). Moreover, the TF activity of Mef2a has been directly implicated in multiple metabolic and mitochondrial functions, such as glucose metabolism (via Glut4 expression) (Gong et al., 2011; Smith et al., 2008), lipid metabolism (via Acc and Cyp7a1 expression) (Clark et al., 2013; Zhang et al., 2024), and mitochondrial biogenesis (via PGC‐1α expression) (Shen et al., 2025).

Another TF of note is Myc, which binds to E‐box sites and is a protooncogene known to play a role in the regulation of cell cycle entry and progression, differentiation, apoptosis, and metabolism (Pelengaris & Khan, 2003; Vernon & Gaston, 2000). Scientific progress in the last two decades has uncovered a multitude of roles and gene targets of Myc both in vivo and in vitro (Morrish & Hockenbery, 2014). During cell‐cycle entry, this TF has been shown to correlate with increases in mitochondrial biogenesis, mitochondrial network interconnectivity, respiration, and substrate availability, likely to meet the steep energetic demand of proliferation. In this study, Myc's inferred transcriptional activity was significantly downregulated at 14‐ and 28‐dpi (Figure 4f); however, the single‐cell data tells a more contextualized story. In only the early temporal groups, Myc's inferred transcriptional activity was upregulated in multiple cell types including endothelial and macrophage. Conversely, in only late temporal groups, Myc was significantly downregulated in a few cell types including satellite and smooth muscle cell types.

More broadly, this study highlights multiple TFs with significantly altered activity throughout the time course, most notably Spi1. This TF is known to be required for the development of both myeloid and lymphoid lineages (Dakic et al., 2007; DeKoter et al., 2002; DeKoter & Singh, 2000). A recent prospective transcriptomic study following VML injury identified Spi1 and Sp1 as potential targets for addressing the chronic inflammation associated with this injury model (Jain et al., 2025). Single‐cell results suggest that the transcriptional signal of Spi1 originates from four cell types: endothelial, dendritic, satellite, and T/NK cells. Notably, Spi1 was not significant in the remaining hematopoietic cell types of monocytes, macrophages, neutrophils, erythroblasts, and B cells. In total, this retrospective study presents multiple potential targets for future interventions such as Hif1a, Tead4, Nr2c2, Mef2a, Myc, and Spi1.

### 4.3 Transcriptome analysis supports VML pathophysiology transition at approximately 2‐week post‐injury

Estimated mean cell proportions in the bulk dataset show that myonuclei and satellite cell populations are immediately altered and stabilized by 14 dpi (Figure 2g, Table S3). Data reported from the authors of the single‐cell study show that in uninjured samples myonuclei and satellite cells occupied 0.5% and 5.4% of the total cell population, respectively. Myonuclei were notably low in the uninjured samples; furthermore, this percentage rose to 2.9% and 3.1% at days 7 and 14 and dropped down to 0.4% and 0.08% at 28 and 42 dpi. These intriguing cell proportions may explain the sparse results from myonuclei. Consistent with the bulk study, satellite cells rose to 6.6% at day 7 post injury and trended to stabilize (2.9%, 4.4%, and 3.0% at 14, 28, and 42 dpi). Also consistent with bulk study results, T/NK cell populations spiked following injury and returned to baseline with time (1.2%, 11.0%, 6.3%, 0.9%, and 0.6% in uninjured, 7‐, 14‐, 28‐, and 42‐dpi samples, respectively).

Among the time points examined in both studies, day 14 post‐VML injury stands out as a critical transitional phase in both the pathophysiology and mitochondrial transcriptome. At this point in a typical regenerative injury, the acute inflammatory phase is resolving while biological processes of the tissue remodeling phase are ramping up; processes such as collagen deposition, angiogenesis, and myogenesis. Previous physiological work examining VML injury has described the remaining tissue at 14 days as being plagued with sustained inflammation (Greising et al., 2018; Larouche et al., 2022), altered collagen deposition leading to fibrotic scarring (Greising et al., 2018; Hoffman et al., 2022), and the beginning of injury‐induced secondary denervation (Hoffman et al., 2024; Sorensen et al., 2021). Results from both the bulk and single‐cell RNA sequencing analysis showed an inflection point at 14 dpi that supports that the transitional phase of VML pathophysiology is captured by the mitochondrial transcriptome regulation as well. For example, there were far fewer downregulated DEGs at 14 days (*n* = 61) compared to 7 days (*n* = 427) (Figure 2) for bulk‐RNA, and a shift in direction from upregulation to downregulation of GSEA pathways for the single‐cell RNA (Figures 4 and 5). Understanding the factors that contribute to the transition phase of the repair can help inform the design of treatment strategies. For example, several studies do not start rehabilitation until the 1–2 week post‐injury time point, and although not explicitly stated, perhaps as an effort to allow the pathophysiology to stabilize (Habing et al., 2024; Motherwell et al., 2023; Nicholson et al., 2024; Quarta et al., 2017; Washington et al., 2021; Ziemkiewicz et al., 2023). The mitochondrial transcriptome time course data can also be used to develop milestones for evaluating successful rehabilitation, for example, by mitigating or even reversing trends in the early downregulation phase to better prepare the remaining muscle for future health. One could hypothesize that the prevention of the early downregulation phase could be accomplished noninvasively with a pharmacologic approach, for example, as utilized previously (Southern et al., 2019). Future approaches should consider leveraging the mitochondrial transcriptome DEGs and TFs for potential targets.

### 4.4 Further considerations

This retrospective study was restricted to existing datasets that were designed with completely uninjured controls and had a time course component. Using the now established timeline, future studies could analyze the mitochondrial transcriptome regulation in studies that utilized spatiotemporal RNA sequencing approaches (Larouche et al., 2023), and/or focused on a single time point comparison of treatment strategies [e.g., in pigs (Greising et al., 2017) and rodents (Roberts et al., 2023)]. These may further advance our understanding of the mitochondrial transcriptome's role in the VML pathophysiology.

## CONFLICT OF INTEREST STATEMENT

The authors declare no conflicts of interest.

## ETHICS STATEMENT

This study was a retrospective analysis of previously published datasets and did not involve any new data collection from humans or animals. Therefore, institutional review board (IRB) and institutional animal care and use committee (IACUC) approvals were not required.

## Supporting information


Figure S1.

Table S1.

Table S2.

Table S3.

Table S4.

Table S5.

Table S6.

Table S7.

Table S8.

Table S9.

Table S10.
