# Supplementary material for: Retrospective transcriptomic analysis indicates temporal dysregulation of mitochondrial genes and metabolic pathways after volumetric muscle loss injury
Source: Physiol Rep. 2025 Nov 2;13(21):e70612. doi: 10.14814/phy2.70612 (PMC12580409; doi:10.14814/phy2.70612)
Supplement: Supplementary file 1 — Figure S1. [file PHY2-13-e70612-s004.zip › Figure S1.docx]

**Figure S1.** Rat Principal Component Analysis (PCA) plots by cell type following differential gene expression analysis by experimental group. Red indicates uninjured samples and blue indicates injured samples.
