# Supplementary figures and images for: Retrospective transcriptomic analysis indicates temporal dysregulation of mitochondrial genes and metabolic pathways after volumetric muscle loss injury
Source: Physiol Rep. 2025 Nov 2;13(21):e70612. doi: 10.14814/phy2.70612 (PMC12580409; doi:10.14814/phy2.70612)

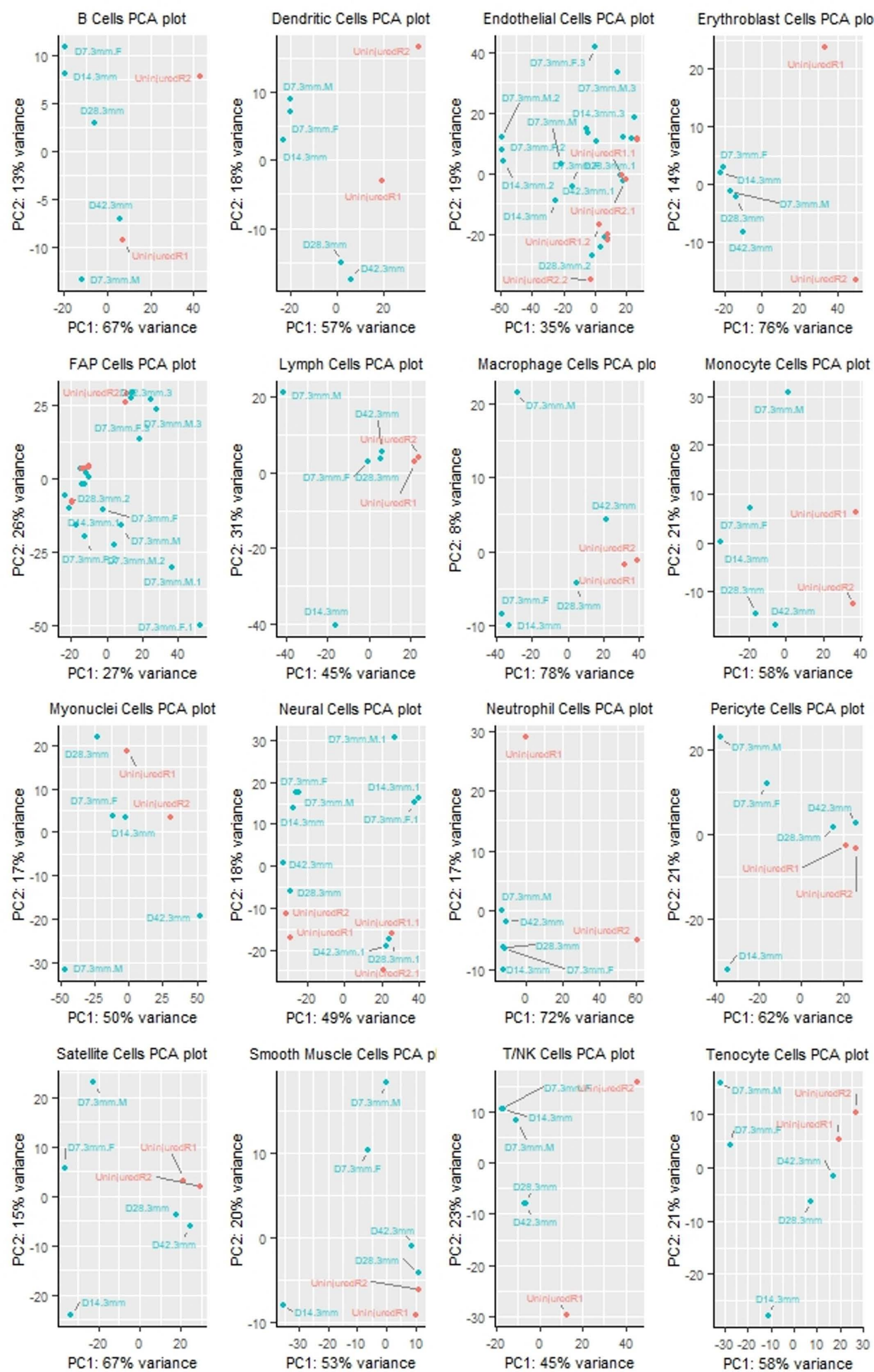

Supplement: Supplementary file 1 — Figure S1. [file PHY2-13-e70612-s004.zip › PHYSREP-2025-05-416-T-s01.pdf]
